# Preventing visual feedback of the moving limb during grasping preferentially activates parietofrontal premotor areas

**DOI:** 10.1016/j.neuroimage.2026.122004

**Published:** 2026-05-14

**Authors:** Nathan A. Baune, Noah Marchal, Scott H. Frey, Kenneth F. Valyear

**Affiliations:** a Department of Rehabilitation Medicine, Emory University, Atlanta, USA; b Department of Psychological Sciences, University of Missouri, Columbia, USA; c School of Psychology, Bangor University, 355 Brigantia Building, Bangor, Wales LL57 2AS, UK; d Bangor Imaging Unit, Bangor University, Bangor, UK

**Keywords:** Grasping, State estimation, Sensorimotor control, Proprioception, Sensory feedback, Forward models, fMRI, Posterior parietal cortex, Premotor cortex

## Abstract

Accurate control of grasping relies on the brain’s ability to estimate the position and configuration of the arm and hand, integrating sensory feedback with motor-based predictions derived from a copy of the movement command. Visual feedback of the moving limb plays an important role in calibrating these internal estimates. When visual feedback is limited, the brain must rely more heavily on proprioception and motor-based predictions. In this study, we use fMRI to test for cortical regions that are preferentially involved in grasp control when visual feedback of the moving limb is selectively prevented. Twenty healthy participants perform reaching-to-touch and reaching-to-grasp actions with and without visual feedback. Critically, target objects are always visible, fixed to illuminate only their surface properties. This contrasts with prior studies that remove vision of both the limb and the target, thereby confounding non-visual control with increased working-memory demands. Our findings reveal a distributed network of posterior parietal and frontal premotor areas that are preferentially active for grasping when visual feedback of the moving limb is unavailable. We interpret these results as reflecting an important role for these areas in maintaining an internal representation of the state of the arm and hand for online grasp control.

## 1. Introduction

Accurate control of goal-directed movement depends on the brain’s ability to estimate the current state of the body in space. Because sensory signals are delayed and noisy, the sensorimotor system cannot rely on sensory input alone; instead, it combines afferent feedback with prior expectations and an internal copy of motor commands—efference copy—to maintain an internal estimate of the body’s configuration and position in space over time (Desmurget and Grafton, 2000; Li et al., 2023; Shadmehr et al., 2010; Wolpert et al., 1995). This process is known as state estimation, providing a continuously updated representation of limb position necessary for planning, monitoring, and adjusting movements as they unfold.

A central component of this process is proprioception: the sensory information arising from receptors in muscles, joints, and skin that signals limb position and movement. Proprioceptive inputs provide ongoing information about limb posture and movement that is critical for planning and executing actions and for manipulating objects (Delhaye et al., 2018; Proske and Gandevia, 2012). When grasping, successful performance depends on maintaining an accurate estimate of the moving hand relative to the target as it approaches, preshapes, and closes on the object (Connolly and Goodale, 1999; Jakobson and Goodale, 1991; Jeannerod, 1984). Evidence from studies of chronic upper-limb sensory deafferentation—where touch and proprioception are profoundly impaired despite relatively intact motor output—shows that loss of somatosensory feedback markedly disrupts grasp control (Gentilucci et al., 1994; Hermsdorfer et al., 2008; Miall et al., 2019). Grasping is slower and more variable, with exaggerated hand opening and less stable preshaping; impairments are especially evident during the later stages of prehension, when finger closure and final adjustments must be coordinated with object contact. This work underscores the importance of proprioception for state estimation and online grasp control.

Visual feedback plays a crucial role in calibrating internal estimates of limb state, especially during actions that demand high spatial precision, such as reaching and grasping (Brenner and Smeets, 2025; Jakobson and Goodale, 1991; Jeannerod, 1996; Prablanc et al., 1986; Schettino et al., 2003; Schlicht and Schrater, 2007; Sivakumar et al., 2021). When visual feedback of the moving limb is unavailable, state estimation must rely more heavily on non-visual sources of information—proprioception and forward-model predictions derived from efference copy (Wolpert et al., 1995). In this study, we identify the cortical regions important for grasp control when visual feedback of the moving limb is unavailable, selectively increasing reliance on proprioceptive and motor-based signals for state estimation and online control.

A well-characterized network of parietofrontal brain regions supports the planning and execution of reaching-to-grasp movements. In non-human primates, two parallel pathways have been described (Fattori et al., 2017; Galletti et al., 2003; Rizzolatti and Matelli, 2003). The dorsolateral pathway connects the anterior intraparietal area (AIP), at the anterior extent of the intraparietal sulcus, with the ventral premotor area F5, in the arcuate sulcus. The dorsomedial pathway connects three regions of the superior parietal cortex—medial intraparietal area (MIP), on the medial bank of the intraparietal sulcus; area V6A, on the anterior bank of the parieto-occipital sulcus; and area 5 of the superior parietal lobule—with the dorsal premotor area F2. Evidence from multiple domains suggests that a similar functional organization exists in humans (Castiello, 2005; Culham and Valyear, 2006; Grafton, 2010; Jeannerod et al., 1995; Valyear, 2018). These areas form the core parietofrontal grasp network. Together with the primary motor and somatosensory cortices, cerebellum, and basal ganglia, this network transforms visual, proprioceptive, and motor-related signals into coordinated movement commands for the skillful control of the arm and hand for object grasping.

The cortical control of grasping in humans has been studied extensively using functional MRI. A common approach involves comparing grasping of real objects against control conditions where the hand is moved to touch those same objects, but without preshaping the digits. This contrast is thought to isolate brain areas involved in configuring the hand and digits for grasping, transforming object features into movement commands for successful prehension. Studies using this approach reliably identify areas of the core parietofrontal grasp network introduced above (Castiello, 2005; Culham et al., 2006, 2003; Frey et al., 2005).

Many of these previous studies examine object grasping without visual feedback by removing vision of both the moving limb and the target object (for a recent meta-analysis, see Sartin et al., 2023). In these studies, participants view the object before movement onset, but the action itself is performed in darkness. This experimental design feature is often described as a control strategy to eliminate visual motion confounds—ensuring that grasp > touch contrasts do not identify brain areas sensitive to differences in visual motion driven by digit movements during grasping. However, this approach introduces a major interpretative limitation. Without continuous vision of the target, participants must rely on memory of its position, size, shape, and orientation. Resultant fMRI activity for grasping may reflect either the processes involved in online control and state estimation—including increased reliance on proprioceptive inputs and motor-based predictions—or visual working memory, different explanations that are difficult to disentangle.

Other imaging studies have addressed these confounds by keeping targets visible while manipulating visual feedback of the moving effector (for e.g., Bernier and Grafton, 2010; Grefkes et al., 2004; Ogawa et al., 2006). These studies are important because they show that posterior parietal and premotor regions flexibly support action according to available sensory information, including contexts in which proprioceptive signals become more heavily weighted. However, these studies use reaching or cursor-control tasks that differ fundamentally from grasping, which requires multi-digit preshaping and the selection of stable grasp points. It remains unclear which brain areas are involved in the control of real object grasping when visual feedback of the moving limb is limited.

In addition, most previous fMRI studies of grasping use simple geometric shapes as target objects, with minimal variation across trials. Under such conditions, participants can form stereotyped grip configurations that become increasingly automatic with repetition. Reliance on memorized motor programs may further complicate efforts to link prior results to state estimation and online control.

Here, we address these limitations using a novel experimental method designed to isolate the contributions of non-visual state estimation during grasping under conditions that increase reliance on proprioceptive and motor-predictive signals while avoiding visual working-memory confounds. Visual feedback of the moving limb is selectively removed while the target object remains continuously visible. This is achieved using custom-built objects embedded with internal lighting, fixed to illuminate only the object’s surface. As a result, visual information about the object’s shape, size, and orientation remains available, while visual feedback of the moving limb and surrounding workspace is selectively removed.

To further reduce reliance on memorized motor strategies, we use a diverse set of graspable shapes with carefully controlled affordances. Sixteen custom-designed *Blake shapes* were used, each with a limited number of stable grasp points and distinctive surface contours that require fine-grained digit positioning (Blake, 1992; Goodale et al., 1994). Object shape and orientation varied across trials, increasing task complexity and minimizing the potential for automatized responses. Under these conditions, successful grasp performance depends on real-time estimation of the limb’s position and configuration relative to the visible object. Memory-based strategies would be insufficient.

By maintaining continuous access to visual object features while selectively eliminating visual feedback of the moving limb, our design enables a more precise test of non-visual state estimation during grasp control. We identify brain regions that are preferentially active when grasping must be guided with reduced visual information about the moving hand, and therefore must depend more heavily on proprioceptive and forward-model signals for estimating limb state.

## 2. Methods

### 2.1. Participants

Twenty-two healthy right-handed adults were recruited for this study. Two participants were excluded from the analysis due to hardware malfunctions during data collection, resulting in a final sample of twenty individuals (22–28 years of age.; 13 female). All participants had normal or corrected-to-normal vision and no history of neurological disorders. The study was approved by the University of Missouri Institutional Review Board in accordance with the Declaration of Helsinki, and written informed consent was obtained from all participants.

A sample size of N = 20 is consistent with the extant neuroimaging literature; a recent meta-analysis reports a mean sample size of 15.2 participants (range 6—31) across 37 studies of grasping (Sartin et al., 2023).

### 2.2. Apparatus and stimuli

#### 2.2.1. Presentation apparatus

**Fig. 1.**
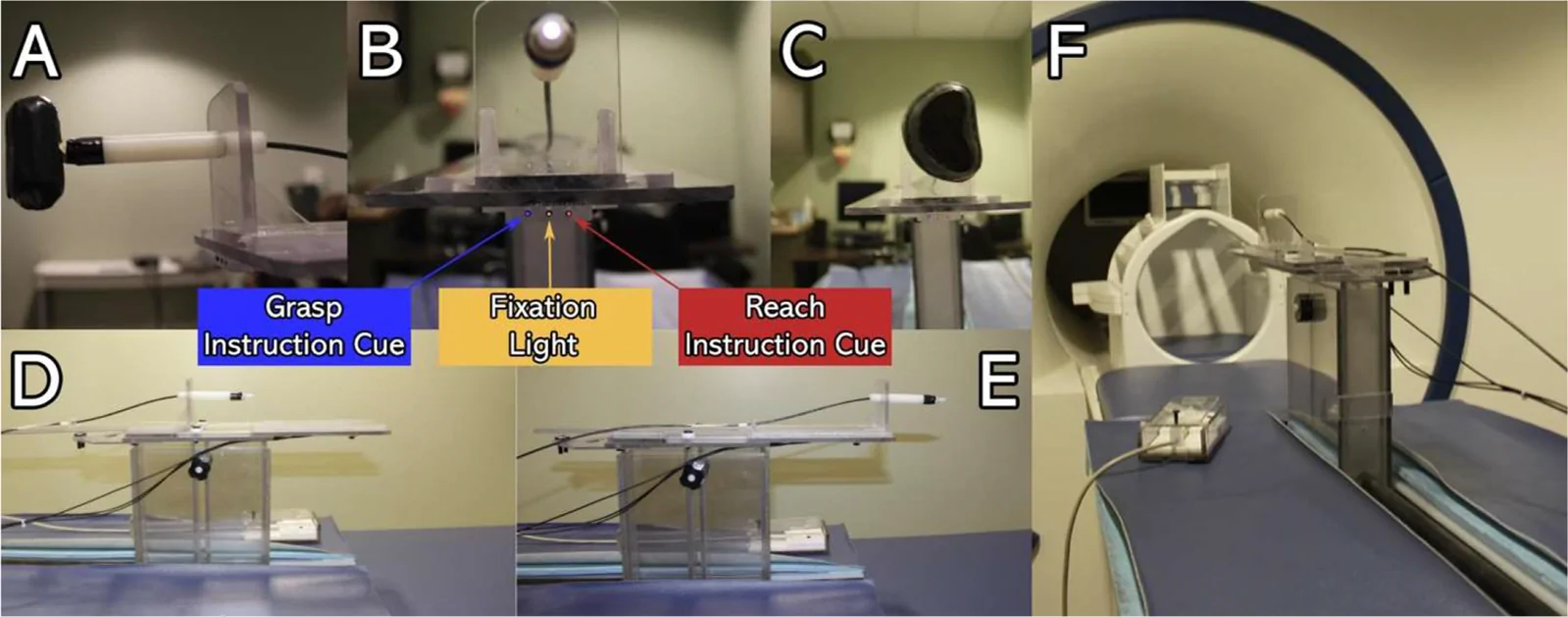
Object presentation apparatus. **(A)** Target object attached to the illumination fiber mounting post. **(B)** Front view of the presentation apparatus without an attached object. Directly beneath the illumination fiber mounting post are the fixation light and two instruction cue lights. **(C)** Front view of the presentation apparatus with an attached object, as seen from the participant’s point of view. **(D)** The illumination fiber mounting post is attached to a slide mechanism used by the experimenter to exchange objects between trials. The slide is shown retracted (i.e., in the position used to swap objects). **(E)** The slide fully extended (i.e., in the position used for participant interaction). **(F)** The presentation apparatus on the mock scanner bed. A mirror affixed to the top of the head coil allows participants to see the workspace. Once positioned with their head in the coil, the presentation apparatus is adjusted so that objects are within arm’s reach at a comfortable distance from the participant’s waist.

We designed an MR-safe object presentation apparatus to present grasp targets approximately 10 cm above the participant’s waist (Fig. 1). The apparatus used a slide mechanism for interchanging objects between trials. Participants viewed the object workspace through a double mirror attached to the Siemens head coil.

Four fiber optic cables connected to an Arduino Leonardo with super-bright colored LEDs were used to manipulate object and workspace visibility (white light), provide a fixation point (yellow light), and display instruction cues (blue and red lights). A single white LED was used to illuminate the grasp target via an optical fiber positioned inside the object. Each object was fabricated from transparent material, with non-exposed surfaces frosted to diffuse transmitted light. Reflective foil was applied to internal surfaces to enhance light reflection, then sealed in opaque black tape to prevent light leakage, leaving only the clear rim exposed. This treatment produced a soft, localized glow that illuminated the object surface while keeping the participant’s limb and surrounding workspace in darkness. Pilot testing confirmed that the hand was not visible under these conditions.

#### 2.2.2. Grasp/Touch targets

We created 16 uniquely shaped target objects based on designs by Blake (1992). These asymmetrical objects with smoothly bounded contours provide limited optimal contact points for a pincer grasp, challenging participants to select appropriate—i.e., stable—grasp points on each trial (Goodale et al., 1994), minimizing the potential for memorized or automatic responses. The objects ranged in size from 3—5.5 cm on a given axis. An example object is shown in Fig. 1, and images of all 16 objects are provided in Supplementary Figure S1.

#### 2.2.3. Scanner bore illumination

Two 10 mm diameter optical fibers attached to the bore wall above the participant (at 45- and 135-degree points) transferred light from super-bright white LEDs housed in the MR control room. This setup allowed for selective control of the entire workspace and limb visibility.

### 2.3. Experimental design and procedure

#### 2.3.1. Training session

In the same session, prior to scanning, participants completed a training session in a mock scanner to acclimate to the environment and ensure task comprehension. The mock scanner replicated the lighting conditions of the actual scanner. Participants completed 16 trials while the experimenter provided immediate feedback. The experimenter monitored whether movements were smooth and did not noticeably transfer to the head or shoulder, whether the correct action was performed according to the task instructions (reaching-to-touch or reaching-to-grasp, as cued by the instruction LED), and whether central fixation was maintained. Feedback was given whenever head movement or task errors were observed. If needed, an additional 16 trials were completed. All participants demonstrated stable head position and accurate task performance before proceeding to the main experiment.

#### 2.3.2. MR procedure

**Fig. 2.**
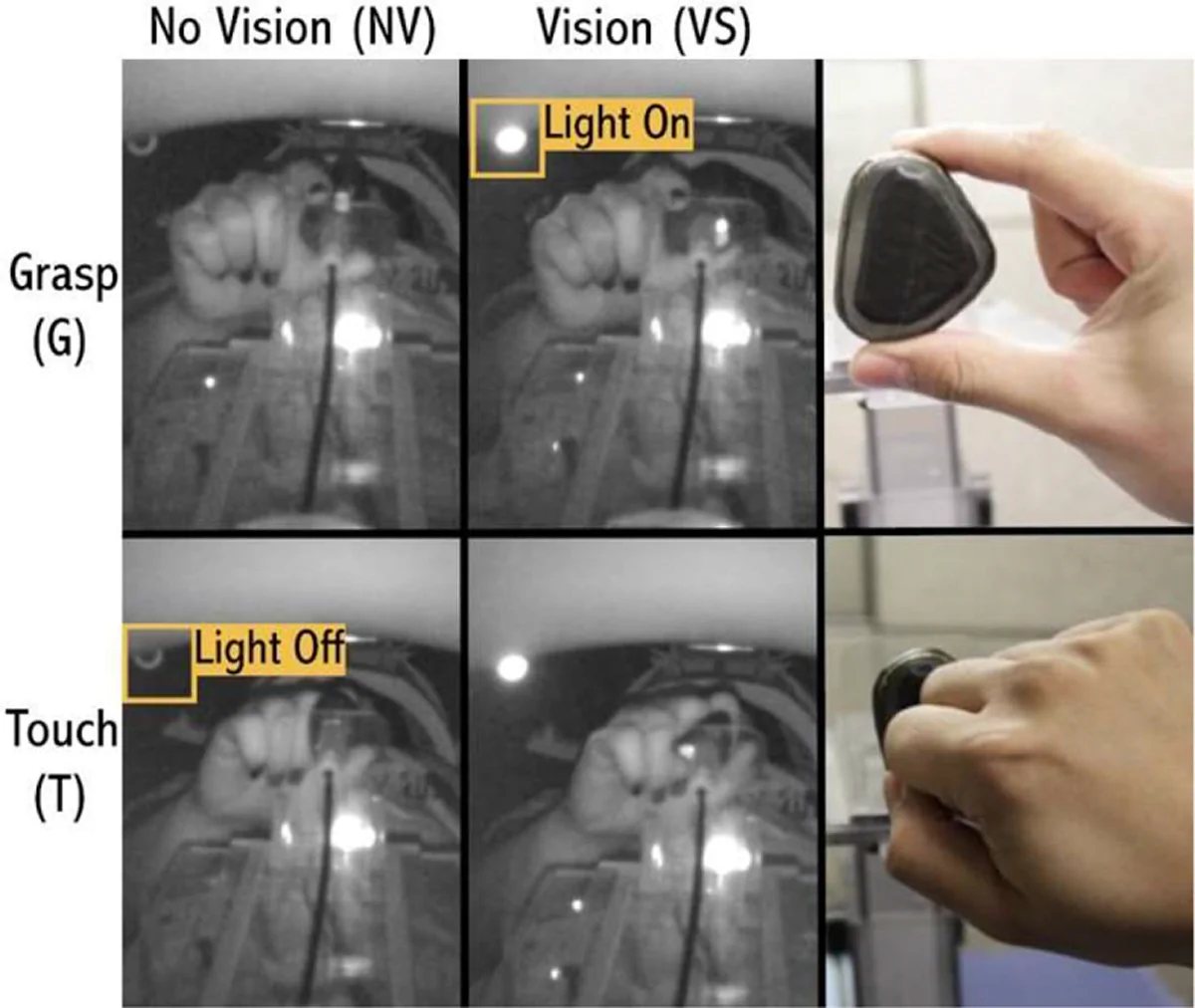
Experimental Design. G = “Grasp,” T = “Touch,” NV = “No visual feedback,” VS = “with visual feedback”. The far-right images show the pincer grasp (top) and closed fist touch (bottom), as demonstrated to participants during task training.

The experiment employed a two-by-two factorial design: Grasp (G) vs. Touch (T), and Vision (VS) vs. No-Vision (NV) of the hand/arm (Fig. 2; Supplementary Videos S1—S4). Participants were positioned in the scanner with the presentation apparatus adjusted for comfortable reaching. A strap secured the upper arms to prevent shoulder movement, and foam padding around the head minimized head motion.

**Fig. 3.**
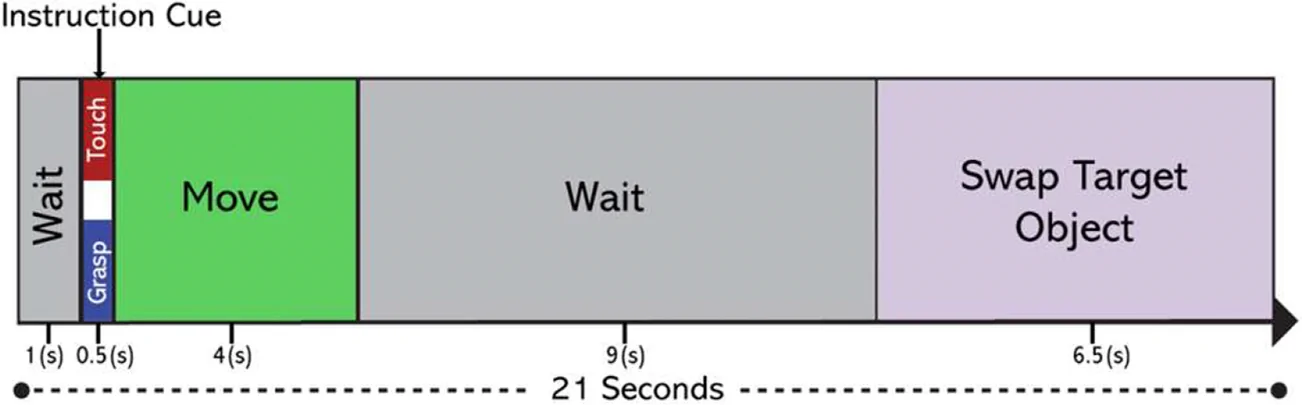
Trial structure and timing. Timeline showing the sequence of events within a single trial. Participants began each trial fixating on a yellow light, followed by presentation of the instruction cue (red for grasp, blue for touch), then object illumination with or without workspace illumination. Movement execution began upon object illumination, with participants returning to the starting position after task completion. Total trial duration was 21 s.

Fig. 3 illustrates the trial structure and timing. Each trial began with participants fixating on a yellow light in an otherwise dark workspace. A cue light (red or blue) indicated whether to grasp or touch the object. After a short delay, the object was illuminated. In half the trials, workspace illumination accompanied object illumination. Participants initiated the instructed action upon object illumination and returned to the starting position after completion. An auditory cue, provided via headphones to the experimenter only, signaled the experimenter to change the object for the next trial.

Participant performance was monitored using an MR-compatible in-bore infrared camera system (MRC Systems GmbH). An experimenter monitored the video feed online from the control room and logged trial-by-trial errors. All sessions were then re-reviewed offline by a second experimenter to confirm error classification.

This slow event-related design provides specific advantages when studying real actions in the scanner (Culham, 2006). Any head displacement caused by arm movements occurs in real time, whereas the BOLD response associated with movement planning and control is delayed. By spacing trials widely, possible transient movement-related artifacts do not overlap with the delayed haemodynamic response from preceding trials, reducing the likelihood that head motion contaminates the neural signal of interest.

Participants completed 8 runs, each comprising 16 trials (4 per condition, randomized within each run). Total experiment duration was approximately 2.5 h.

#### 2.3.3. Imaging parameters

Imaging was performed on a 3-Tesla Siemens TIM Trio MRI scanner with a conventional 8-channel birdcage head coil. The T1-weighted anatomical images were collected using a multiplanar rapidly acquired gradient echo (MP-RAGE) pulse sequence: time to repetition (TR) = 1920 ms; time to echo (TE) = 2.92 ms; flip angle = 9; matrix size = 256 × 256; field of view (FOV) =256 mm; 176 contiguous sagittal slices; slice thickness = 1 mm; in-plane resolution = 1 mm × 1 mm. Auto Align Scout and True FISP sequences were executed before the start of each functional run to ensure that slices were prescribed in the same positions across runs. Functional MRI volumes were collected using a T2*-weighted single-shot gradient-echo-planar imaging (EPI) acquisition sequence: TR =3000 ms; TE =30 ms; flip angle =84; matrix size =64 × 64; FOV = 200 mm; slice thickness = 3 mm; in-plane resolution = 3.125 mm × 3.125 mm; acceleration factor (integrated parallel acquisition technologies, iPAT) = 2 with generalized auto calibrating partially parallel acquisitions (GRAPPA) reconstruction. Lastly, a gradient echo field map scan was acquired for distortion correction of the EPI images.

### 2.4. Data processing and analysis

#### 2.4.1. Preprocessing

Structural and functional MRI data were preprocessed and analyzed using FSL v.5.0.8 (Smith et al., 2004). Non-brain structures were removed using BET. Head movement was processed using MCFLIRT (Jenkinson et al., 2002). Functional data underwent high-pass temporal frequency filtering (cutoff: 0.01 Hz) and were aligned to high-resolution anatomical volumes using FLIRT. Data were then transformed to standard MNI-152 space using FNIRT nonlinear registration and spatially smoothed with a 6 mm FWHM Gaussian kernel.

#### 2.4.2. First-level analysis

Single-subject analyses were conducted using fixed-effects general linear models (GLMs) in FEAT v.6.0, with FILM applied to correct for serial correlations. Each run underwent intensity normalization using “grand mean scaling” to enable valid between-run and between-subject comparisons.

GLMs included independent explanatory variables (EVs) for each condition and their temporal derivatives. Condition-specific EVs were modeled as fixed 4-s rectangular wave functions corresponding to the period of target-object illumination, when actions were performed, convolved with a double-gamma hemodynamic response function. Temporal derivatives were included to accommodate minor timing variability in the hemodynamic response. Additional nuisance regressors included error trials (identified from video monitoring), motion parameter estimates from MCFLIRT, and single-volume spike regressors identified using *fsl_motion_outliers* (default settings). This tool computes the root mean square (RMS) intensity difference between each volume and a reference volume and flags outliers using the standard box-plot cutoff (75th percentile + 1.5 × interquartile range). Error-trial EVs were modelled in the same way as condition EVs: as 4-s rectangular wave functions corresponding to the target illumination/action period, convolved with the double-gamma hemodynamic response function.

#### 2.4.3. Whole-brain analyses

Group-level voxel-wise analyses were implemented using random-effects models in FLAME 1. All statistical maps were thresholded at z > 2.3, corresponding to an uncorrected voxel-level p < 0.01, and surviving clusters were identified using a cluster-size corrected threshold of p < 0.05. Throughout, references to “significant activation” refer to this thresholding method.

A functional inclusion mask was first defined by combining the outcomes of the following four contrasts using an OR operation: (i) G-VS > rest; (ii) G-NV > rest; (iii) T-VS > rest; (iv) T-NV > rest. Any voxel showing significant activation in at least one of these contrasts was included in the mask. The purpose of this step was to increase sensitivity for subsequent analyses by restricting the search volume to voxels responsive to the task.

Two contrasts were then evaluated. First, we tested for a main effect of Grasp by contrasting Grasp > Touch, collapsed across visual feedback conditions. This contrast was used to identify regions showing higher responses for grasping, expected to identify known areas of the parietofrontal grasp network. Identifying this network served as a validation step and reference point for interpreting interaction effects.

Second, we tested the Action by Visual Feedback Interaction, expressed as (G–NV > T–NV) > (G–VS > T–VS). This was the central contrast of interest, used to identify regions where grasp-related responses are selectively increased when visual feedback of the moving limb was prevented.

#### 2.4.4. Cluster analysis: qualifying response patterns

We next performed a cluster analysis to interrogate the response profiles within a select number of regions that were identified by the Action by Visual Feedback Interaction. The goal of this analysis was to determine whether the condition-specific response patterns within these regions were consistent with our theoretical framework.

The interaction contrast identifies voxels where the difference between Grasp and Touch depends on visual feedback, but it does not specify *how* this dependence arises. For example, a cluster could reach significance because responses are greater for Touch than Grasp under full-vision conditions (T–VS > G–VS), with little or no Grasp-selective response in the no-vision condition. Such a pattern would be incompatible with our theoretical framework—these effects could not be interpreted as increased demands on state estimation and online control.

We focused on clusters located in posterior parietal cortex, premotor cortex, primary sensorimotor cortex, and the cerebellum—regions for which we had *a priori* predictions. Importantly, these clusters were not newly defined ROIs. They were exactly the clusters identified by the whole-brain interaction contrast (at z > 2.3, cluster-corrected at p < 0.05). The Jülich atlas (Eickhoff et al., 2005) was used solely as an anatomical reference. No additional voxels were added, and no small-volume or ROI-restricted statistical inference was performed. All statistical inference was based solely on the whole-brain corrected interaction contrast; subsequent analyses examined only the pattern of responses within those already significant clusters.

Condition-wise %-BSC values were extracted and entered into a repeated measures ANOVA using the ‘ezANOVA’ function from the ‘ez’ package (Lawrence, 2006) for R (CRAN Team, 2018), and post-hoc pairwise comparisons were performed using one-sided paired *t*-tests (G-NV > T-NV and G-VS > T-VS) with the ‘pairwise.t.test’ function. Multiple comparisons were corrected using the Holm adjustment method. Confidence intervals were calculated using the ‘wsci’ function from the ‘papaja’ package (Aust and Barth, 2022), employing the Cousineau-Morey method for within-subjects designs (Cousineau, 2005; Morey, 2008).

To further evaluate functional-anatomical correspondence, we conducted a supplementary cross-reference analysis of peak locations against independent atlas resources. For cortical peaks, we compared the original Jülich-based labels with the HCP-MMP1.0 parcellation (Glasser et al., 2016); for cerebellar peaks, we additionally consulted the Diedrichsen probabilistic cerebellar atlas (Diedrichsen et al., 2009). The resulting atlas correspondences are provided in Supplementary Table S1.

## 3. Results

Motion correction estimates indicate that transient arm-movement-related head motion was minimal (see Supplementary Table S2). Few task errors were observed (19/2560 trials; 0.74%; max =4 errors for any single participant; max = 2 errors for any single run). Example clips of each error type are provided in Supplementary Videos S5—S9.

All reported results are thresholded at Z > 2.3 (voxel-level p < 0.01; cluster-size corrected at p < 0.05).

### 3.1. Whole-brain analyses

#### 3.1.1. Main effect of grasp

**Fig. 4.**
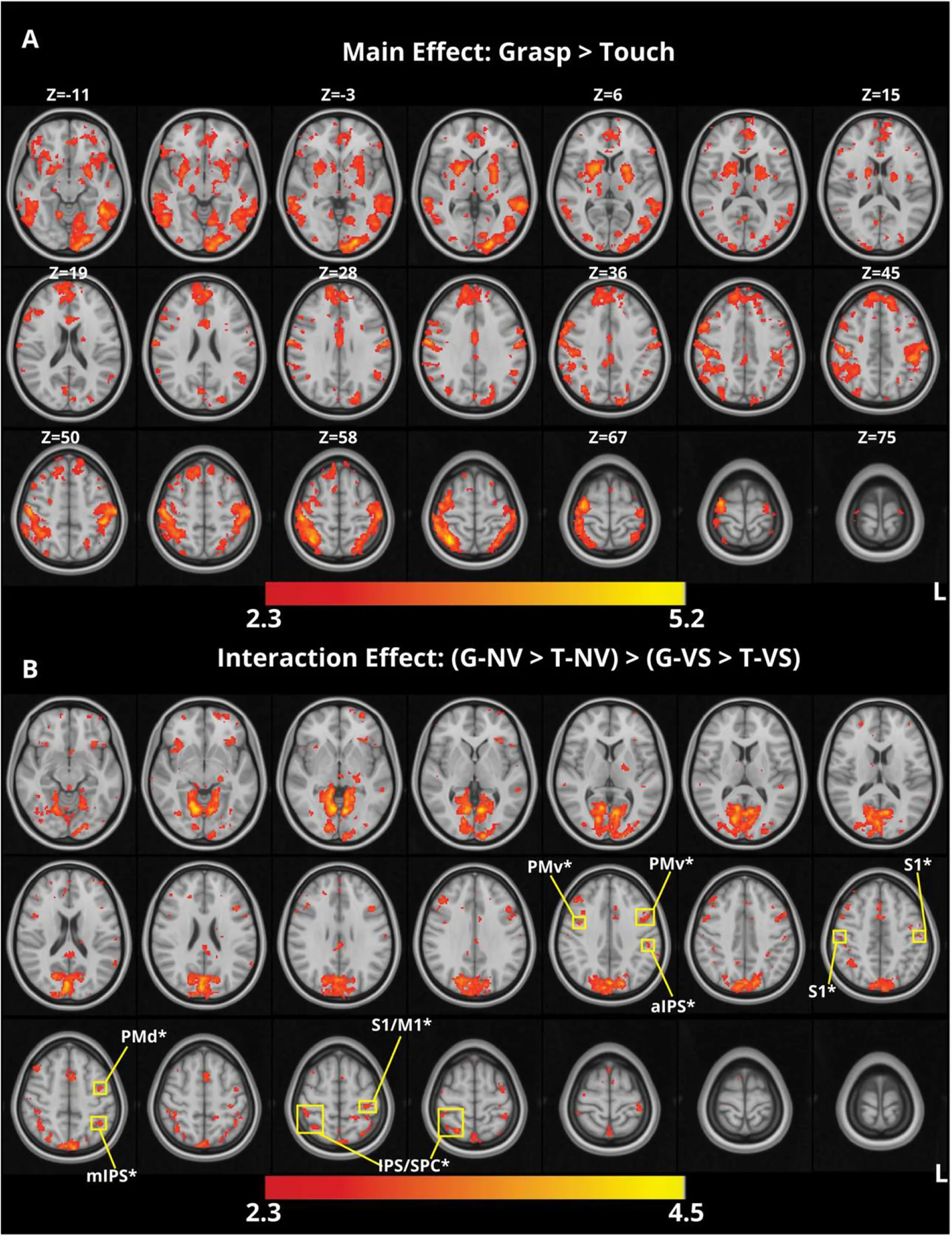
Whole-Brain Voxel-Wise Results. **(A)** Areas showing significantly greater fMRI responses for grasping compared to touch, collapsed across visual feedback conditions, identified by the contrast Grasp > Touch. **(B)** Areas showing a significant interaction between Action type and Visual Feedback, identified by the contrast (G-NV > T-NV) > (G-VS > T-VS). Statistical maps are thresholded at z > 2.3 (voxel-level p < 0.01; cluster-corrected at p < 0.05). Abbreviations: PMv (ventral premotor cortex); PMd (dorsal premotor cortex); aIPS (anterior intraparietal sulcus); mIPS (mid-intraparietal sulcus); IPS/SPC (intraparietal/superior parietal cortex); S1 (primary somatosensory cortex); S1/M1 (primary sensorimotor cortex); POJ (parieto-occipital junction). Asterisks denote clusters that do not survive a stricter threshold of z > 3.1 (p < 0.001, cluster-corrected at p < 0.05).

As a first step, we conducted a whole-brain voxel-wise contrast of Grasp > Touch, collapsed across visual conditions. This was done to evaluate whether our paradigm activates the expected network of grasp-related brain regions. The results agree with previous fMRI studies, revealing robust activation for object grasping within a distributed parietofrontal network, including bilateral anterior intraparietal, superior parieto-occipital, and the ventral and dorsal premotor cortices (Fig. 4A). These results show the quality of our data and provide a functional reference point for interpreting the interaction effects reported below.

#### 3.1.2. Action by visual feedback interaction

We next tested whether grasp-related responses are modulated by the availability of visual feedback of the hand—our central focus. Our goal was to isolate brain regions preferentially involved in non-visual state estimation and online grasp control: the processes by which the brain monitors and adjusts the position and configuration of the moving limb in real time using proprioceptive and motor-based signals.

To accomplish this, we conducted a whole-brain voxel-wise Action (type) by Visual Feedback Interaction contrast: (G-NV > T-NV) > (G-VS > T-VS). This contrast isolates regions where grasp-related activity is increased under non-visual conditions—where online control must rely more heavily on proprioceptive input and forward-model predictions. These regions are therefore implicated in the internal control processes that support non-visual state estimation for grasp control.

The results reveal widespread activation across posterior parietal and frontal cortices, including key regions of the core parietofrontal grasp network (Fig. 4B). In the hemisphere contralateral to the acting hand (left hemisphere), significant activity is observed in the precentral cortex—likely overlapping with dorsal premotor cortex (PMd)—and in the central sulcus, encompassing primary somatosensory and motor areas (S1/M1) and extending anteriorly into the precentral gyrus. Activation is evident bilaterally along the length of the intraparietal sulcus (IPS), with distinct clusters in anterior and mid-IPS (aIPS, mIPS), dorsomedial foci overlapping with the superior parietal cortex (SPC), and more posterior foci near the parieto-occipital junction (POJ). Anterior IPS and the postcentral gyrus are also active bilaterally, likely overlapping with Brodmann areas 1 and 2 of S1. The ventral precentral cortex—likely including ventral premotor area (PMv)—and medial frontal areas, including the cingulate, supplementary motor area, and pre-supplementary motor area are also identified, bilaterally. Although not shown here, activity is also found in the cerebellum (see Fig. 7D/E and Supplementary Figure S2).

Regions outside the canonical grasp network are also identified, including a large swath of activity along the medial occipital cortex. As the focus of this study is on the parietofrontal cortices, we do not further examine the response profiles of these regions.

Supplementary Figure S3 shows the spatial overlap between the main effect of Grasp > Touch (Fig. 4A) and the Action × Visual Feedback Interaction (Fig. 4B), highlighting both shared and distinct regions identified by each contrast.

As a robustness check, Supplementary Figure S4 shows the Action × Visual Feedback interaction thresholded at z > 3.1 (p < 0.001, cluster-corrected at p < 0.05); clusters in Fig. 4B that do not survive this more stringent thresholding are marked with an asterisk.

#### 3.1.3. Cluster analysis: qualifying response patterns

While the Action (type) by Visual Feedback Interaction contrast is used to identify brain regions with selectively enhanced responses for grasping without visual feedback, the resulting effects may arise from other condition differences. Specifically, regions may be identified because they show greater activity for Touch than Grasp with visual feedback (T-VS > G-VS)—i.e., the ‘righthand side’ of the interaction contrast. In such cases, grasping without vision may not elicit stronger responses than touch without vision, which would be inconsistent with our interpretation—namely, that these effects reflect increased metabolic demands for object grasping due to greater reliance on non-visual body-state estimation and online movement control.

To address this, we examine condition-wise percent BOLD signal change (%-BSC) response profiles within regions identified by the interaction contrast. Response profiles showing statistically greater activation for Touch than Grasp with visual feedback (T-VS > G-VS) would raise concerns, suggesting that the interaction is driven by a pattern inconsistent with our a priori interpretative framework.

#### 3.1.4. Posterior parietal cortex

**Fig. 5.**
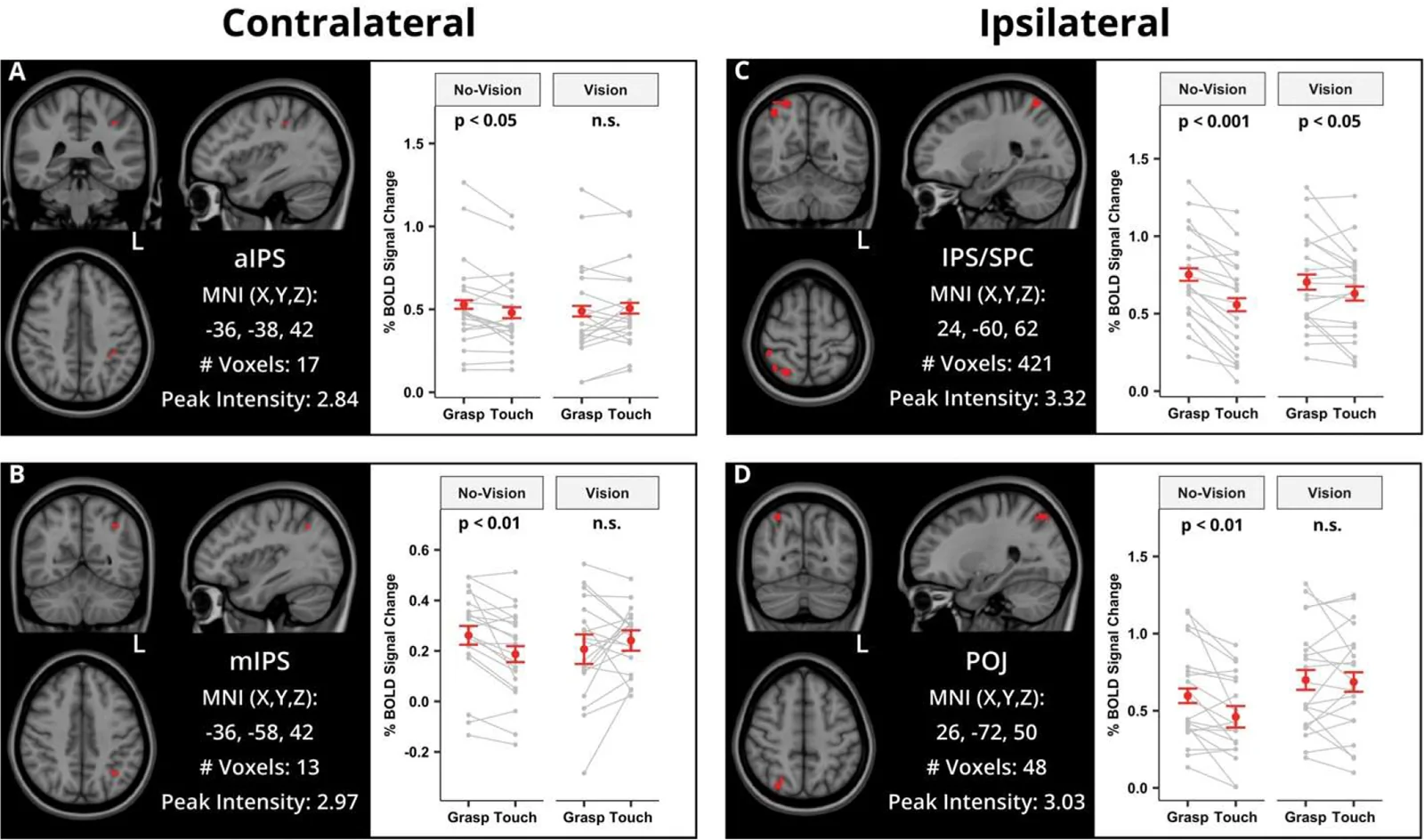
Posterior Parietal Cortex. Percent BOLD signal change (%-BSC) data extracted from areas within the posterior parietal cortex identified by the interaction contrast: (G-NV > T-NV) > (G-VS > T-VS), thresholded at z > 2.3 (voxel-level p < 0.01; cluster-corrected at p < 0.05). Scatter plots show condition-wise %-BSC values from each area, with individual participant values shown in gray and group means ± 95% confidence intervals shown in red. Statistical results for pairwise comparisons are indicated. Brain area abbreviations as in Fig. 4.

Contralateral to the grasping hand, two distinct clusters are identified within the IPS. One is located at the anterior end of the sulcus, consistent with the reported location of grasp-selective area AIP in prior fMRI studies (Culham et al., 2003; Frey et al., 2005; Valyear et al., 2019) (Fig. 5A). The second lies approximately midway along the IPS (Fig. 5B). Both regions show statistically reliable Grasp > Touch effects in the no-vision condition, and no significant differences between Grasp and Touch when full visual feedback is available. This pattern suggests that these IPS regions are selectively recruited for grasp control under conditions of increased reliance on non-visual feedback.

On the ipsilateral side, a large cluster of activity is observed within the superior parietal cortex, spanning anterior to posterior positions along the medial bank of the IPS (Fig. 5C). The response profile shows significant Grasp > Touch effects for *both vision and no-vision conditions*; however, these effects are more robust in absence of visual feedback, driving the significant interaction.

A separate, more posterior cluster is located near the parieto-occipital junction (POJ; Fig. 5D). This region shows a statistically reliable Grasp > Touch effect in the no-vision condition, accompanied by a significant main effect of visual condition (F = 6.09, p =0.02), driven by greater activation under full-vision compared to no-vision conditions. Grasp and Touch responses do not differ when visual feedback is available.

This response profile suggests that the cell populations within this area are highly visually responsive, yet not preferentially involved in grasp control under typical viewing conditions. Grasp-selectivity emerges only when visual feedback of the moving limb is absent. This suggests a role for the POJ in non-visual body-state estimation and online movement control, while also highlighting its robust responsivity to visual input—being more strongly activated when both the moving limb and the surrounding environment are visible.

#### 3.1.5. Premotor cortex

**Fig. 6.**
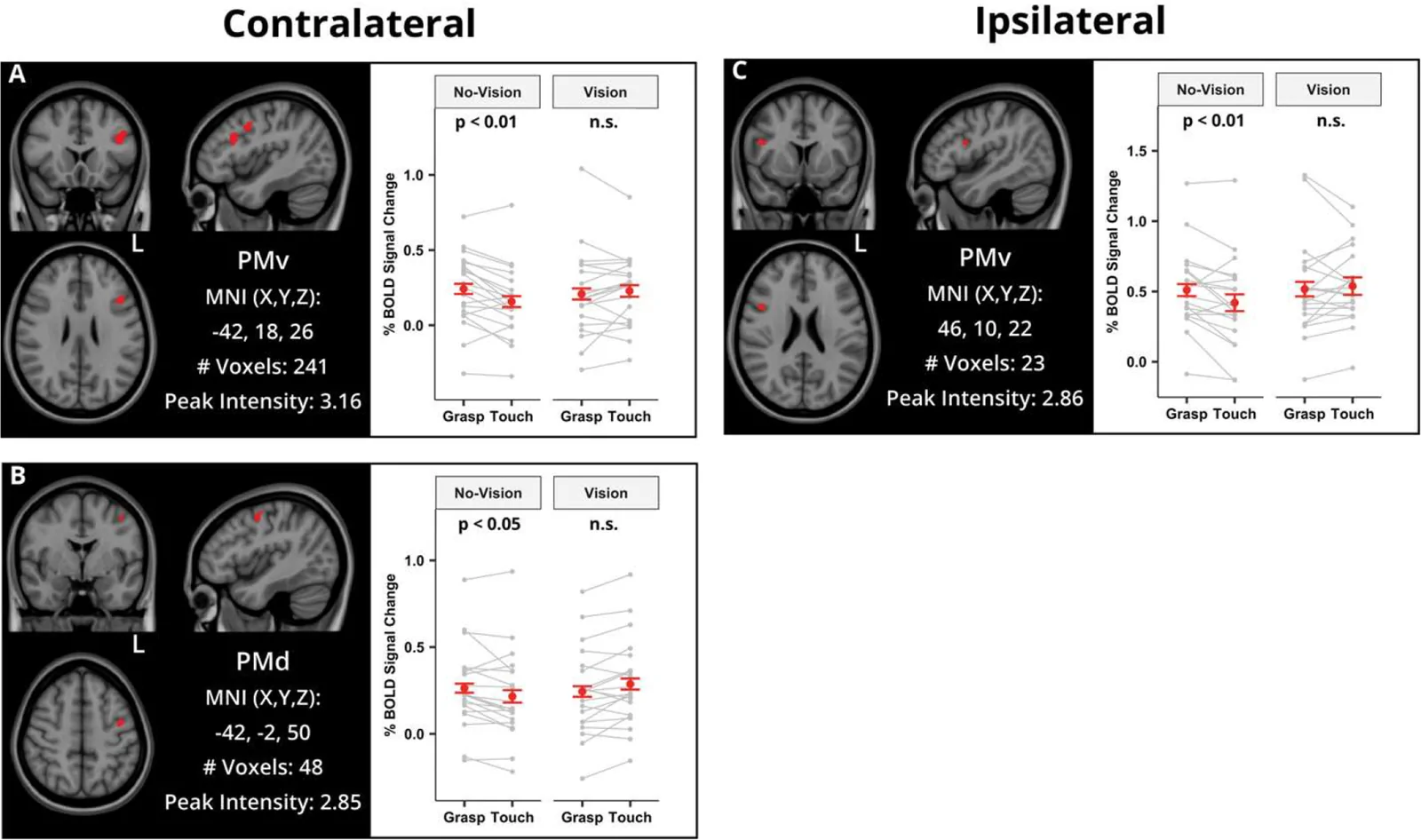
Premotor Cortex. Percent BOLD signal change (%-BSC) data extracted from areas within the ventral and dorsal premotor cortex identified by the interaction contrast: (G-NV > T-NV) > (G-VS > T-VS), thresholded at z > 2.3 (voxel-level p < 0.01; cluster-corrected at p < 0.05). Scatter plots show condition-wise %-BSC values from each area, with individual participant values shown in gray and group means ± 95% confidence intervals shown in red. Statistical results for pairwise comparisons are indicated. Brain area abbreviations as in Fig. 4.

Interaction effects are observed bilaterally within ventral aspects of the precentral sulcus, including parts of the middle and inferior frontal gyri (Fig. 6A and 6C). These regions likely correspond with the putative human homologue of monkey area F5, ventral premotor cortex (PMv), sometimes reported in prior fMRI studies of grasping in humans (e.g., Valyear et al., 2019). On the contralateral side, a distinct cluster of activity is identified within the dorsal precentral sulcus, likely overlapping with the dorsal premotor cortex (PMd) (Fig. 6B). The location of this activation is consistent with previous human fMRI studies reporting grasp-selective PMd (for meta-analysis, see Valyear et al., 2017). All three regions show a statistically reliable Grasp > Touch effect in the no-vision condition, and no significant difference between conditions when visual feedback is available.

The results within ventral and dorsal premotor areas align with our interpretative framework. The interaction effects reflect greater activity for Grasp over Touch without visual feedback and are not driven by greater activation for Touch over Grasp with visual feedback.

#### 3.1.6. Primary sensorimotor cortex and the cerebellum

**Fig. 7.**
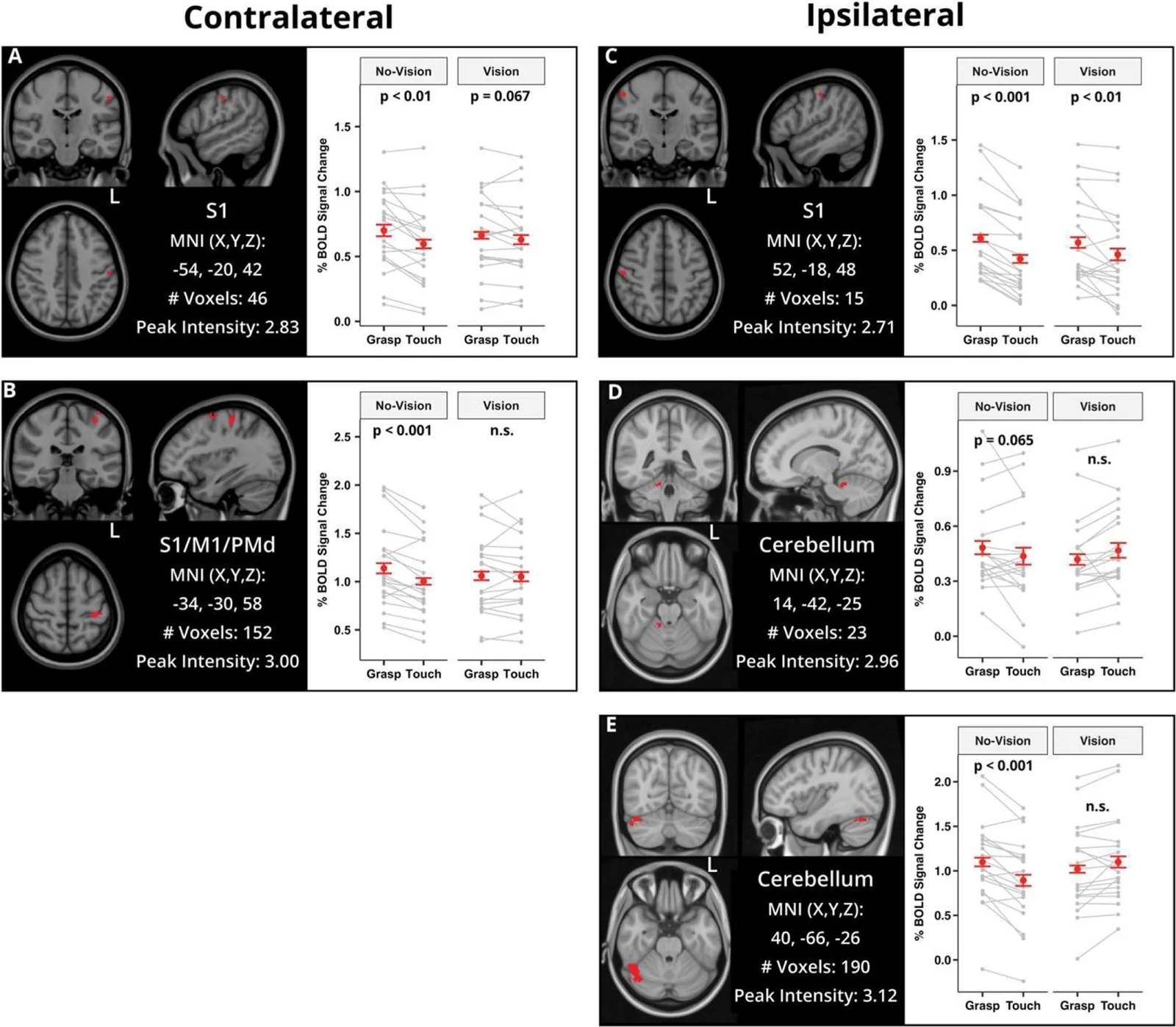
Primary sensorimotor cortex and the cerebellum. Percent BOLD signal change (%-BSC) data extracted from areas within primary sensorimotor cortex and the cerebellum identified by the interaction contrast: (G-NV > T-NV) > (G-VS > T-VS), thresholded at z > 2.3 (voxel-level p < 0.01; cluster-corrected at p < 0.05). Scatter plots show condition-wise %-BSC values from each area, with individual participant values shown in gray and group means ± 95% confidence intervals shown in red. Statistical results for pairwise comparisons are indicated. Brain area abbreviations as in Fig. 4. The S1/M1 area includes parts of the precentral gyrus, possibly overlapping with PMd.

Anterior parietal cortex is identified bilaterally, likely corresponding to primary somatosensory (S1) areas BA1 and BA2 (Fig. 7A and 7C). On both sides, this activity overlaps with the postcentral gyrus. The response patterns are similar across hemispheres. On the contralateral side, S1 shows a statistically reliable Grasp > Touch effect in the no-vision condition, and a non-significant trend in the same direction under full-vision conditions. Ipsilaterally, significant Grasp > Touch effects are observed for *both vision and no-vision conditions*; however, these effects are more robust without visual feedback, driving the significant interaction.

Contralateral to the acting hand, a large, contiguous cluster spanning the central and precentral sulci is also identified (Fig. 7B). The portion within the central sulcus likely corresponds to the primary somatosensory and motor cortices (collectively, S1/M1), while the dorsal precentral component may overlap with PMd. The response profile across this region shows significantly stronger responses for Grasp than Touch in the no-vision condition, with no reliable Grasp > Touch differences under full-vision conditions.

Numerous clusters of activity are also observed within the cerebellum. Fig. 7D shows a cluster found in the anterior ipsilateral cerebellum (lobules I-IV), close to the location of grasp-selective responses reported in previous fMRI studies (Sartin et al., 2023; Valyear et al., 2019). Notably, however, the response profile in this area is inconsistent with our interpretive framework: Grasp > Touch effects during no-vision do not reach significance, and responses during full vision are numerically greater for Touch than Grasp. In other words, the interaction observed in this area reflects only weak, nonsignificant Grasp > Touch effects when acting without visual feedback accompanied by relatively high Touch responses under full-vision conditions. This pattern is difficult to interpret and illustrates the importance of conducting these additional cluster-wise response profile analyses.

Otherwise, cerebellar activation is distributed predominantly across posterior lobules bilaterally. As an example, Fig. 7E shows a second ipsilateral cluster located laterally within Crus I (lobule VII). The response profile in this cluster is consistent with our interpretive framework, showing greater activity for Grasp over Touch without visual feedback and no significant differences between Grasp and Touch with full vision available.

All other cerebellar clusters and their response profiles are presented in Supplementary Figure S2. These include a contralateral counterpart to the Crus I cluster in Fig. 7E (Figure S2E), additional clusters in bilateral lobules VIIb (Figure S2B, S2F) and VIIIa (Figure S2D, S2G), and a small cluster spanning contralateral Crus I/II (Figure S2C). Similar to the anterior cluster in Fig. 7D, a small posterior cluster in contralateral Crus I (Figure S2A) also showed a response pattern inconsistent with our interpretive framework.

## 4. Discussion

This study isolates cortical regions that respond preferentially to object grasping in the absence of visual feedback of the moving limb. By maintaining continuous vision of the grasp target while selectively removing vision of the moving limb, we increase demands on non-visual sources of information—proprioception and motor-based predictions—for online grasp control. Our results reveal a distributed network of regions showing grasp-selective responses specifically under these conditions, including areas within posterior parietal and dorsal and ventral premotor cortices. We consider these results with respect to models of state estimation, in which efference copy and proprioceptive inputs are used to estimate limb state in the absence of visual feedback.

Previous fMRI investigations of grasping without visual feedback have prevented vision of both the moving limb and the target object (for a meta-analysis, see Sartin et al., 2023). Grasping under these conditions must rely on memory of the object’s position, size, shape, and orientation. This makes it difficult to interpret corresponding fMRI responses as linked to state estimation and online control. Instead, such activity may reflect visual working memory. Indeed, when grasping is performed after a time delay, the maintenance of visual object information involves intraparietal and premotor cortices (Fiehler et al., 2011), with some studies also showing involvement of both early and higher-level visual areas (Monaco et al., 2017; Singhal et al., 2013).

In the current study, we kept the target object visible while preventing vision of the moving limb. Our results showing preferential responses to grasping without visual feedback of the moving limb cannot be attributed to visual working memory of object properties such as size, shape, or orientation. Further, because our target objects have complex surface contours affording limited stable grasp configurations and were varied from trial-to-trial, automatized grasp responses were unlikely. These conditions place high demands on real-time grasp control.

By testing for an interaction between action type (Grasp, Touch) and visual feedback (Vision, No-vision), we isolate regions where grasp-selective responses are increased when vision of the moving limb is selectively withheld. While we interpret these effects as reflecting increased demands on internal control mechanisms involved in non-visual state estimation for online grasp control, we first consider whether they might also be explained by performance-based differences in movement patterns or somatosensory feedback.

### 4.1. Performance-based interpretations

Grasping without vision of the moving limb may have changed how the limb was moved in our study, and perhaps these changes could account for the interaction effects we observe. Previous studies using motion capture methods reveal changes in the kinematics of reaching-to-grasp movements when visual feedback of the moving limb is selectively removed while maintaining vision of the target object (Churchill et al., 2000; Connolly and Goodale, 1999; Rand et al., 2007; Schettino et al., 2003; Sivakumar et al., 2021). Increased movement durations—particularly during the deceleration phase—and changes in the timing and extent of hand opening and closing are often reported. Further, without visual feedback, the digits may undergo more frequent or prolonged postural adjustments near and after object contact, altering not only movements but also proprioceptive and touch-based inputs during grasp stabilization.

Such behavioural changes provide a plausible alternative account for our findings. Regions identified by the interaction contrast may reflect increased neural activity driven by altered movement patterns and/or somatosensory inputs, rather than internal state estimation processes specifically.

According to this performance-based account, however, one might reasonably expect such regions to also show greater activation for Grasp compared to Touch under full-vision conditions. Grasping involves longer movements, distinct and more complex digit and hand preshaping, and additional tactile inputs from the digits on and after object contact. In other words, interaction effects that reflect performance-based differences may be expected to show grasp specificity—Grasp > Touch—for *both* no-vision and full-vision conditions.

Two regions showed response patterns consistent with this—superior parietal cortex (SPC; Fig. 5C) and S1 (Fig. 7A/C). Both regions showed statistically greater responses for Grasp over Touch for both visual feedback conditions, with the interaction driven by more pronounced differences in the no-vision condition. The SPC is known to play a vital role in the online control of reach-to-grasp movements (Archambault et al., 2009; Chen et al., 2009; Glover et al., 2005; Grea et al., 2002; Tunik et al., 2008), while S1 encodes touch and proprioceptive signals from the digits (Delhaye et al., 2018; Francis et al., 2000; Gardner et al., 2007). An account of these results as driven by condition differences in movement patterns, proprioceptive and/or touch-based inputs is acceptable.

Nevertheless, the interaction effects observed in these areas may also reflect contributions to state estimation for grasp control. Proprioceptive inflow is itself one of the core signals from which limb state is estimated. Thus, increased responses in SPC and S1 may reflect condition differences in both movement-related somatosensory and proprioceptive drive, and increased demands on the use of these signals for state estimation during grasp control. Both interpretations are viable and are not mutually exclusive. The condition-specific response profiles in several other regions are less readily explained by a sensorimotor performance-based account and more strongly support the state-estimation interpretation we detail below.

### 4.2. State estimation

State estimation refers to the process by which the brain maintains an updated internal representation of the body’s configuration in space to support ongoing motor control. Spatially distinct regions of the anterior and posterior intraparietal and superior parietal cortices—including the parieto-occipital junction—as well as the dorsal and ventral premotor cortex show response selectivity for grasping without visual feedback of the moving limb. In the absence of visual feedback, greater reliance on proprioception and motor-based predictions increases demands on state estimation. We suggest that the observed activity in these areas reflects these increased demands, highlighting their critical contributions to state estimation and online grasp control.

This interpretation—particularly our findings in medial superior parietal areas—aligns with the model proposed by Fattori et al. (2017), in which monkey area V6A, a central node of the dorsomedial reach-to-grasp pathway, acts as a comparator for state estimation. Neurons in area V6A integrate visual, proprioceptive, and motor-related signals, showing complex response patterns that vary with the availability of visual feedback. Cells tuned to different movement parameters during reaching show enhanced or suppressed activity in the presence of visual feedback (Bosco et al., 2010). These response properties suggest that V6A maintains an updated estimate of limb configuration for the planning and control of reaching and grasping.

According to this model, area V6A provides sensory information about the state of the upper limb to the dorsal premotor area F2, which in turn provides motor-related signals—including an efference copy of ongoing motor commands—to V6A. This bidirectional communication enables V6A to act as a state estimator, comparing predicted and actual limb states during reach-to-grasp planning and execution (Bosco et al., 2010; Ciavarro et al., 2013; Fattori et al., 2017).

Area V6A has dense, reciprocal connections with the dorsal premotor area F2, and both areas show response properties consistent with the encoding of reaching and grasping movements (Fattori et al., 2004, 2010; Hendrix et al., 2009; Raos et al., 2004; Stark et al., 2007). V6A is also densely connected with area MIP (Gamberini et al., 2009)—another critical node in the dorsomedial pathway involved in goal-directed arm control (Andersen et al., 2014; Snyder et al., 1997)—and with superior parietal area PEc (Gamberini et al., 2009), from which V6A receives somatosensory information about arm position (Bakola et al., 2010; Breveglieri et al., 2006).

The putative functional human homolog of monkey area V6A is found in the precuneus, on the anterior wall of the parieto-occipital sulcus (Pitzalis et al., 2015; Vesia and Crawford, 2012). Our interaction effects include this area, as well as other key nodes of the dorsomedial pathway—superior parietal cortex, MIP, and the dorsal premotor cortex. Previous fMRI studies of reaching and grasping consistently implicate these areas (Castiello, 2005; Cavina-Pratesi et al., 2010; Sartin et al., 2023; Valyear, 2018), and converging evidence from lesion and brain stimulation studies reveals their causal involvement in the planning and control of reaching and grasping in both humans (Andersen et al., 2014; Ciavarro et al., 2013; Grea et al., 2002; Vesia and Crawford, 2012; Vesia et al., 2010) and monkeys (Battaglini et al., 2002; Galletti et al., 2003).

Our results suggest that these parietal and premotor regions contribute to state estimation for grasp control by integrating proprioceptive and motor-based signals, consistent with previous studies implicating these same areas in the flexible integration of sensory information for movement control. For example, using fMRI repetition suppression, Bernier and Grafton (2010) show that human posterior parietal cortex—including areas of the superior parietal lobule, intraparietal sulcus, and parieto-occipital junction—and the dorsal premotor cortex flexibly determine the reference frame for reaching depending on available sensory information, representing motor goals differently depending on whether targets are defined visually or proprioceptively. Their results align with computational models proposing that the brain simultaneously maintains multiple reference frames and weights them based on sensory context to optimize movement control (McGuire and Sabes, 2009; Sober and Sabes, 2005). Our findings—revealing enhanced grasp-selective responses within these same parietal and premotor areas when visual feedback of the moving limb is limited—support the idea that these regions contribute to state estimation by dynamically integrating sensory inputs to maintain an updated, task-dependent estimate of limb position and posture.

These conclusions are also consistent with neuropsychological evidence showing that damage to the superior parietal cortex disrupts the maintenance of an accurate internal representation of limb state (Wolpert, Goodbody, et al., 1998).

Alongside the dorsomedial pathway, our results also identify interaction effects within the anterior intraparietal and ventral premotor cortices—key regions of the dorsolateral pathway for prehension (Fattori et al., 2017; Jeannerod, 1996; Murata et al., 2000; Schaffelhofer and Scherberger, 2016; Valyear, 2016). These areas specify hand shape according to object properties and action goals, as well as monitor and update these specifications as movements unfold (Rice et al., 2006; Tunik et al., 2007).

Rapid corrective adjustments made in flight—during ongoing movements—depend on predictive state estimation via forward models (Desmurget and Grafton, 2000; Scott, 2012). These in-flight adjustments are impaired after TMS to anterior intraparietal cortex (Rice et al., 2007, 2006; Tunik et al., 2005). Given the predominant motor coding of primate ventral premotor area F5 (Murata et al., 1997; Raos et al., 2006; Schaffelhofer and Scherberger, 2016; Spinks et al., 2008; Umilta et al., 2007), the ventral premotor cortex may provide motor-related signals to the anterior intraparietal cortex. Together, these areas appear to integrate motor and sensory signals with action goals to generate forward models of the evolving state of the hand (Tunik et al., 2007).

Our results and interpretations also align with evidence implicating the cerebellum as critical for state estimation (Desmurget and Grafton, 2000; Diedrichsen et al., 2007; Kawato, 1999; Miall et al., 2007; Wolpert, Miall, et al., 1998). We observe several spatially distinct patches of cerebellar activity—predominantly within the posterior cerebellum, including bilateral Crus I (lobule VII) and lobules VIIb and VIIIa—showing elevated responses for grasping under reduced visual feedback, consistent with our interpretation of increased demands on internal control mechanisms.

### 4.3. Proprioception

Proprioception is a core contributor to the state-estimation processes discussed above, and the present manipulation necessarily increases reliance on this source of information. Some aspects of our findings therefore fit particularly well with an interpretation that places proprioceptive processing in the foreground, even though our design does not isolate it from motor-based prediction or performance-related changes in movement and somatosensory feedback.

Our interaction effects identify bilateral activity in S1, centred around the postcentral gyrus. This location is consistent with involvement of higher-order anterior parietal fields, including area 2, which contains both cutaneous and proprioceptive responses and is thought to support the integration of these signals for object-related hand function (Delhaye et al., 2018; Iwamura, 1998). The bilateral character of these effects is also compatible with the tendency for receptive fields to become larger and, at more integrative stages of somatosensory processing, sometimes bilateral. These anterior parietal fields project onward into dorsal-stream parietal systems linked to sensorimotor planning and manual action, including posterior parietal regions involved in reaching and grasping (Delhaye et al., 2018). Against this backdrop, the increased responses we observe in postcentral, intraparietal, superior parietal, and premotor regions are compatible with the view that grasping without visual feedback of the hand places greater demands on the cortical processing and integration of proprioceptive information for limb-state estimation and online control.

This interpretation is also broadly consistent with evidence that skilled manual actions depend critically on non-visual sensory information, including proprioception (Gentilucci et al., 1994; Jeannerod et al., 1984; Miall et al., 2019). Taken together, these considerations suggest that the present results should be interpreted with explicit appreciation of proprioception as a key component of non-visual state estimation during grasp control.

### 4.4. Predictive coding

An alternative interpretation of our results can be drawn from predictive-coding theories of motor control. In both predictive-coding and state-estimation frameworks, the brain maintains internal models that predict the sensory consequences of actions. While state estimation specifies how sensory feedback combines with prior expectations and motor commands to generate internal models of body state, predictive coding specifies how ongoing comparisons between predicted and actual sensory signals drive the updating of these models (Johansson and Flanagan, 2009; Li et al., 2023; Shadmehr et al., 2010; Wolpert and Flanagan, 2001). Under reduced visual feedback, prediction-error signals may be larger and more frequent as the hand approaches the target object; our interaction effects may reflect increased processing of prediction errors. Consistent with this, the posterior parietal cortex and cerebellum are critically involved in sensorimotor prediction (Blakemore et al., 2001; Blakemore and Sirigu, 2003; Della-Maggiore et al., 2004; Desmurget and Grafton, 2000; Wolpert, Miall, et al., 1998).

Although we did not record kinematics during scanning, previous behavioural studies that also remove visual feedback of the hand while maintaining vision of the target object report increased movement variability and longer deceleration times (Churchill et al., 2000; Connolly and Goodale, 1999; Rand et al., 2007; Schettino et al., 2003; Sivakumar et al., 2021), consistent with more frequent online corrections. Nonetheless, we view prediction-error signalling as complementary to our state-estimation interpretation, representing an important computational mechanism through which internal models are refined to maintain accurate estimates of limb state during grasp control.

### 4.5. Limitations

In the No-Vision conditions, potential occlusion of the illuminated target object by the moving hand was unavoidable. Our setup, however, ensured that such cues could only arise just before or at the point of object contact. Likewise, if diffuse light from the target object reached the approaching hand—a possibility not observed during piloting—this would have occurred only near contact. While too late to influence online control (Brenner and Smeets, 2025), this information could contribute to state estimation, combining with proprioceptive and tactile signals to correct for spatial trajectory or digit placement errors at object contact.

This possibility does not compromise our results interpretation. Grasping under these conditions still imposes far greater demands on state estimation than grasping with continuous visual feedback, and we do not interpret the interaction effects as reflecting purely nonvisual processing. Instead, we propose that the identified regions integrate all available information to maintain accurate internal estimates of limb state during grasp control.

Measures of movement performance in the scanner would have been valuable. This would enable direct tests of performance-based and prediction-error interpretations; it may have been possible to distinguish fMRI responses more closely related to altered movement patterns or somatosensory inputs from the processing of prediction errors. This represents a limitation of the current study and an important direction for future work.

## 5. Conclusions

Our findings reveal that the posterior parietal and premotor cortices contribute to online grasp control when visual feedback of the moving limb is limited. These regions show enhanced grasp-selective responses under conditions that increase reliance on proprioception and motor-based predictions, consistent with their proposed roles in state estimation. By selectively removing visual feedback of the moving limb while maintaining continuous access to visual information about the target object, our design effectively increases demands on state-estimation without introducing working-memory confounds. Our results support the view that parietofrontal and cerebellar circuits dynamically integrate all available sensory and motor signals to maintain accurate, moment-to-moment control of grasping movements.

## Supplementary Material

1

2

3

4

5

6

7

8

9

10

11

Supplementary material associated with this article can be found, in the online version, at doi:10.1016/j.neuroimage.2026.122004.

## Data Availability

Summary data are available at github.com/NeuroTR0PHY/Baune-Grasp-No-Vision-MRI.
