# Supplementary material for: Preventing visual feedback of the moving limb during grasping preferentially activates parietofrontal premotor areas
Source: Neuroimage. Author manuscript; Available in PMC 2026 Sep 10. (PMC13559751; doi:10.1016/j.neuroimage.2026.122004)
Supplement: Supplementary file 1 [file NIHMS2186693-supplement-7.docx]

**Supplementary Table S1. Cross-reference of interaction-cluster peak locations against independent atlas resources.** For each cluster showing a significant Action × Visual Feedback interaction, the table lists the region label assigned using the Juelich cytoarchitectonic atlas (Eickhoff et al., 2005), peak MNI coordinates (x, y, z), the corresponding parcel from the HCP multi-modal parcellation 1.0 (HCP-MMP1.0; Glasser et al., 2016), and — for cerebellar clusters — the lobule label from the Diedrichsen probabilistic cerebellar atlas (Diedrichsen et al., 2009). HCP-MMP labels were obtained using the FSL-compatible volumetric distribution of the atlas (github.com/mbedini/The-HCP-MMP1.0-atlas-in-FSL).

| **Fig.** | **Original label** | **Peak MNI (x, y, z)** | **HCP-MMP parcel** | **Diedrichsen lobule** | **Note** |
| --- | --- | --- | --- | --- | --- |
| 5a | Left aIPS | −36, −38, 42 | L_AIP | — | Glasser AIP; identified as human putative homologue of macaque area AIP |
| 5b | Left mIPS | −36, −58, 42 | L_IP2 | — | Mid-IPS parcel, between AIP anteriorly and LIP/posterior IPS |
| 5c | Right IPS/SPC | 24, −60, 62 | R_VIP | — | Ventral intraparietal area; multisensory parietal parcel |
| 5d | Right POJ | 26, −72, 50 | R_MIP | — | Medial intraparietal area; Parietal Reach Region |
| 6a | Left PMv | −42, 18, 26 | L_IFJa | — | Inferior frontal junction (anterior); adjacent to classical PMv |
| 6b | Left PMd | −42, −2, 50 | L_FEF | — | Frontal eye field; at border of PMd (area 6d) |
| 6c | Right PMv | 46, 10, 22 | R_IFJp | — | Inferior frontal junction (posterior); adjacent to classical PMv |
| 7a | Left S1 | −54, −20, 42 | L_2 | — | Brodmann area 2; proprioceptive-integration subdivision of S1 |
| 7b | Left S1/M1/PMd | −32, −30, 58 | L_3b | — | Primary cutaneous S1 at peak; large cluster likely spans BAs 3a, 4, 6d |
| 7c | Right S1 | 52, −18, 48 | R_2 | — | Brodmann area 2 (ipsilateral to acting hand); proprioceptive-integration S1 |
| 7d | Right anterior cerebellum | 14, −42, −25 | — | Right lobule I–IV (peak); few voxels from lobule V-predominant | At the boundary of leg/foot (I–IV) and primary hand (V) representations |
| 7e | Right posterior cerebellum | 40, −66, −26 | — | Right Crus I (lobule VII) | Outside primary somatotopic map; implicated in complex hand action and state estimation |
