# Supplementary material for: Preventing visual feedback of the moving limb during grasping preferentially activates parietofrontal premotor areas
Source: Neuroimage. Author manuscript; Available in PMC 2026 Sep 10. (PMC13559751; doi:10.1016/j.neuroimage.2026.122004)
Supplement: Supplementary file 2 [file NIHMS2186693-supplement-8.docx]

**Supplementary Table S2. Head Motion Parameters**. Maximum relative displacement (mm) for each participant across all runs. Maximum relative displacement represents the largest instantaneous (frame-to-frame) head movement across an entire run, indicating the maximum amount the head moved from one time point to the next. Values are expressed in millimeters and represent root-mean-square (RMS) displacement combining both translations and rotations (rotations converted to equivalent linear displacements at the surface of a 50mm radius sphere). Functional voxel size was 3mm³ (slice thickness = 3mm; in-plane resolution = 3.125mm × 3.125mm). Mean = 1.52mm, SD = 0.68mm, Range = 0.42–3.92mm.

| **Subject** | **Mean Relative  Displacement** | **Max Relative  Displacement** |
| --- | --- | --- |
| S1 | 0.13 | 0.67 |
| S2 | 0.14 | 1.24 |
| S3 | 0.11 | 0.46 |
| S4 | 0.11 | 0.75 |
| S5 | 0.12 | 0.33 |
| S6 | 0.15 | 2.40 |
| S7 | 0.19 | 1.10 |
| S8 | 0.10 | 0.92 |
| S9 | 0.29 | 3.92 |
| S10 | 0.11 | 0.89 |
| S11 | 0.06 | 0.16 |
| S12 | 0.08 | 0.34 |
| S13 | 0.06 | 0.19 |
| S14 | 0.09 | 0.39 |
| S15 | 0.08 | 0.32 |
| S16 | 0.11 | 0.44 |
| S17 | 0.11 | 1.35 |
| S18 | 0.08 | 0.32 |
| S19 | 0.08 | 0.35 |
| S20 | 0.09 | 0.37 |
