# Supplementary material for: Preventing visual feedback of the moving limb during grasping preferentially activates parietofrontal premotor areas
Source: Neuroimage. Author manuscript; Available in PMC 2026 Sep 10. (PMC13559751; doi:10.1016/j.neuroimage.2026.122004)
Supplement: Supplementary file 3 [file NIHMS2186693-supplement-9.docx]

Online Supplementary Materials

**Preventing visual feedback of the moving limb during grasping preferentially activates parietofrontal premotor areas**

Nathan A. Baune^1^, Noah Marchal^2^, Scott H. Frey^2^, and Kenneth F. Valyear^3, 4^

1. Department of Rehabilitation Medicine, Emory University, Atlanta, USA.
2. Department of Psychological Sciences, University of Missouri, Columbia, USA.
3. School of Psychology, Bangor University, Bangor, UK.
4. Bangor Imaging Unit, Bangor University, Bangor, UK.

**Supplementary Figures**

**Supplementary Figure S1. Complete set of Blake shape stimuli**. Left: All 16 uniquely shaped target objects based on Blake (1992). Objects have asymmetrical geometries with smoothly bounded contours designed to provide limited optimal contact points for pincer grasp. Right: Front (top), side (middle), and rear (bottom) views of a target object. Grid scale: each square = 1 cm. Objects were fabricated from transparent material, with non-exposed surfaces frosted with sandpaper while maintaining optical clarity at the exposed rim. Reflective foil was applied to internal surfaces to enhance light reflection and prevent light leakage, then sealed in opaque black tape, leaving only the clear rim exposed for controlled light transmission.

**Supplementary Figure S2. Cerebellum**. Percent BOLD signal change (%-BSC) data from additional independent cerebellar clusters not shown in Figure 7 of the main manuscript, identified by the interaction contrast: (G-NV > T-NV) > (G-VS > T-VS), thresholded at z = 2.3 (voxel-level p < 0.01; cluster-corrected at p < 0.05). Scatter plots show condition-wise %-BSC values from each area, with individual participant values shown in gray and group means ± 95% confidence intervals shown in red. Statistical results for pairwise comparisons are indicated.

**Supplementary Figure S3. Overlap between main effect and interaction contrasts.** Spatial overlap between the voxel-wise results of the main effect of Grasp > Touch (blue; see Figure 4A of the main manuscript) and the Action type by Visual Feedback interaction (red; see Figure 4B of the main manuscript). Both shared (yellow) and distinct areas are identified by each contrast. Statistical maps are binarized here, initially thresholded at z = 2.3, (voxel-level p < 0.01; cluster-corrected at p < 0.05), as in Figure 4.

**Supplementary Figure S4. Action × Visual Feedback interaction at a more conservative cluster-forming threshold.** Whole-brain voxel-wise results for the interaction contrast [(G-NV > T-NV) > (G-VS > T-VS)] thresholded at z > 3.1 (voxel-level p < 0.001; cluster-corrected at p < 0.05), shown on axial slices of the MNI152 template (radiological convention, L = left). Warm- and cool-colour maps represent two spatially distinct surviving clusters (max z = 4.74 and 4.66, respectively). At this stricter threshold, significant interaction effects are found within occipital and dorsomedial visuo-parietal cortex, encompassing bilateral early visual areas (V1, V1/V2), V6, and the parieto-occipital (V6/V6A) and posterior superior parietal cortex (SPL; area 7p).
